# Large language model developments

**DOI:** 10.1098/rsos.252099

**Published:** 2026-11-04

**Authors:** Philip Treleaven, Edward Sharkey, Sara Craciun

**Affiliations:** Department of Computer Science, University College London, London, UK

**Keywords:** LLM, LLM developments, RAG

## Abstract

Large language models (LLMs) are generative artificial intelligence (AI) models trained with vast amounts of data and context, and excel at processing, understanding and generating human language, using transformer models to learn statistical relationships between tokens. LLMs are a subset of transformer models, and hence are closely related but not interchangeable. Given the ‘explosion’ of LLM research, the challenge is tracking emerging developments and matching LLMs to applications. This paper reviews LLM developments with the aim of understanding the underlying models’ computational strengths and weaknesses, building on previous work focusing on LLM optimization (Sharkey & Treleaven 2025 Optimising large language models: taxonomy and techniques. *SSRN Scholarly Paper no. 5278456*. Rochester, NY: SSRN). We find that despite their power, LLMs (transformers) face several key challenges. These include: (i) computational complexity and resource requirements—training and deploying large models is extremely costly and slow, and energy consumption is a concern; (ii) understanding long sequences and dependencies—even with enhancements, models can struggle with very long-range dependencies or context lengths beyond their window (e.g. maintaining coherence in a long document), which reflects the more fundamental absence of continual learning; (iii) interpretability and bias—it remains difficult to interpret why a model produces a given output, and models can inadvertently amplify biases present in training data; and (iv) optimization challenges, including fine-tuning for specific tasks without overfitting or losing generality and ensuring efficient inference (latency, the delay before a response is completed) for real-time applications. In summary, this review focuses on architectural, optimization and interpretability developments in LLMs. It does not aim to provide a systematic benchmark comparison, exhaustive implementation details or empirical performance evaluation, which are covered extensively elsewhere. The authors are committed to updating this review aligned to constant changes in the technology; please reach out with any additions.

## 1. Introduction

As large language models (LLMs) and transformer models become more sophisticated, it is increasingly important to understand the various paradigms underpinning their design and functionality. LLMs have garnered significant attention owing to their ability to produce highly coherent and contextually relevant outputs. These models play a pivotal role in tasks such as summarization, translation, sentiment analysis and dialogue systems.

The following sections provide an overview of the prominent LLM technologies. For context, we start with a high-level explanation of the common machine learning (ML) paradigms.

### 1.1. Machine learning paradigms

We first note that these methods are essentially all inductive; what distinguishes them is the supervision signal—supervised learning (labelled ‘gold’ data), self-supervised learning (unlabelled data, as used by transformer LLMs via next-token prediction) and reinforcement learning (a reward signal). In terms of this paper, ML techniques can be divided into:

— **classic ML:** classic ML techniques primarily involve statistical and connectionist models, which rely on feature engineering and discriminative learning. These methods typically require carefully curated datasets and substantial domain knowledge to extract relevant features;
— **generative artifical intelligence (GenAI):** GenAI focuses on creating systems capable of dynamically producing text, images, audio, video, code or other modalities in a manner that appears strikingly human-like. More precisely, generative models learn the distribution of their training data and sample new instances from it, unlike discriminative models that only classify or score existing inputs. LLMs are a subset of GenAI for human-like text and speech;
— *LLM* used to generate text or complete tasks based on their training data and predefined capabilities (e.g. traditional LLMs). In addition, *LLM agents* introduce features to LLMs, such as planning, memory and tool usage, to achieve greater autonomy and handle more complex, multistep tasks; and
— *world models:* GenAI models learn the dynamic behaviours of the real world, including physics and spatial properties. A model that observes its environment and predicts what might happen next, considering its current knowledge and unknown factors that could affect future outcomes [1].

Table 1 shows a simple comparison of the core features of traditional ML and GenAI.

**Table 1.** Comparison of classic ML and GenAI.

feature	classic ML	GenAI
primary function	extract relevant features from curated datasets	generative content creation
focus	natural language processing, etc	generating new content (text, images, etc.)
examples	recommendation and forecasting	text generation, image generation, etc.
key concept	ML, neural networks	LLMs (e.g. NLP), world models

### 1.2. Large language model technologies

Unpacking the key underlying LLM technologies:

— **artificial neural networks (ANNs)**: ANNs are a type of machine learning (ML) process that uses interconnected nodes or neurons in a layered structure ‘inspired’ by the human brain [2]. An ANN uses training examples and learns by adjusting a ‘weight’ associated with each connection linking two neurons [3];
— **deep learning** uses ANNs with multiple layers (i.e. deep neural networks) to analyse data and learn complex patterns [4];
— **natural language processing (NLP)**: as discussed, NLP involves understanding the meaning of text and speech [5]. NLP centres on tokenization, a way of separating a piece of text into smaller units called ‘tokens’. Tokens can be words, characters or subwords; and
— **transformers** are a type of deep learning ANN model used for NLP tasks and can learn long-range dependencies between words in a sentence [6–8].

### 1.3. Tokenization for natural language processing

A crucial first step in NLP is *tokenization*—segmenting text into words, subwords or characters. Comprehensive treatments of word-, character- and sentence-level tokenization appear in chapter 2 of Jurafsky & Martin [9], while state-of-the-art sub-word schemes, such as byte-pair encoding, are covered in [10].

Figure 1 presents a taxonomy of sentence compression methods, from statistical techniques to reinforcement learning. Prominent is deep learning, which uses multiple-layer neural networks. It is broadly divided into recurrent neural networks, convolutional neural networks, graph neural networks and transformers. The ‘dependency parsing’ branch denotes one specific compression technique, not the whole history of semantically motivated NLP, with which transformer methods contrast sharply by replacing explicit linguistic structure with a distributed statistical surface over tokens.

**Figure 1.**
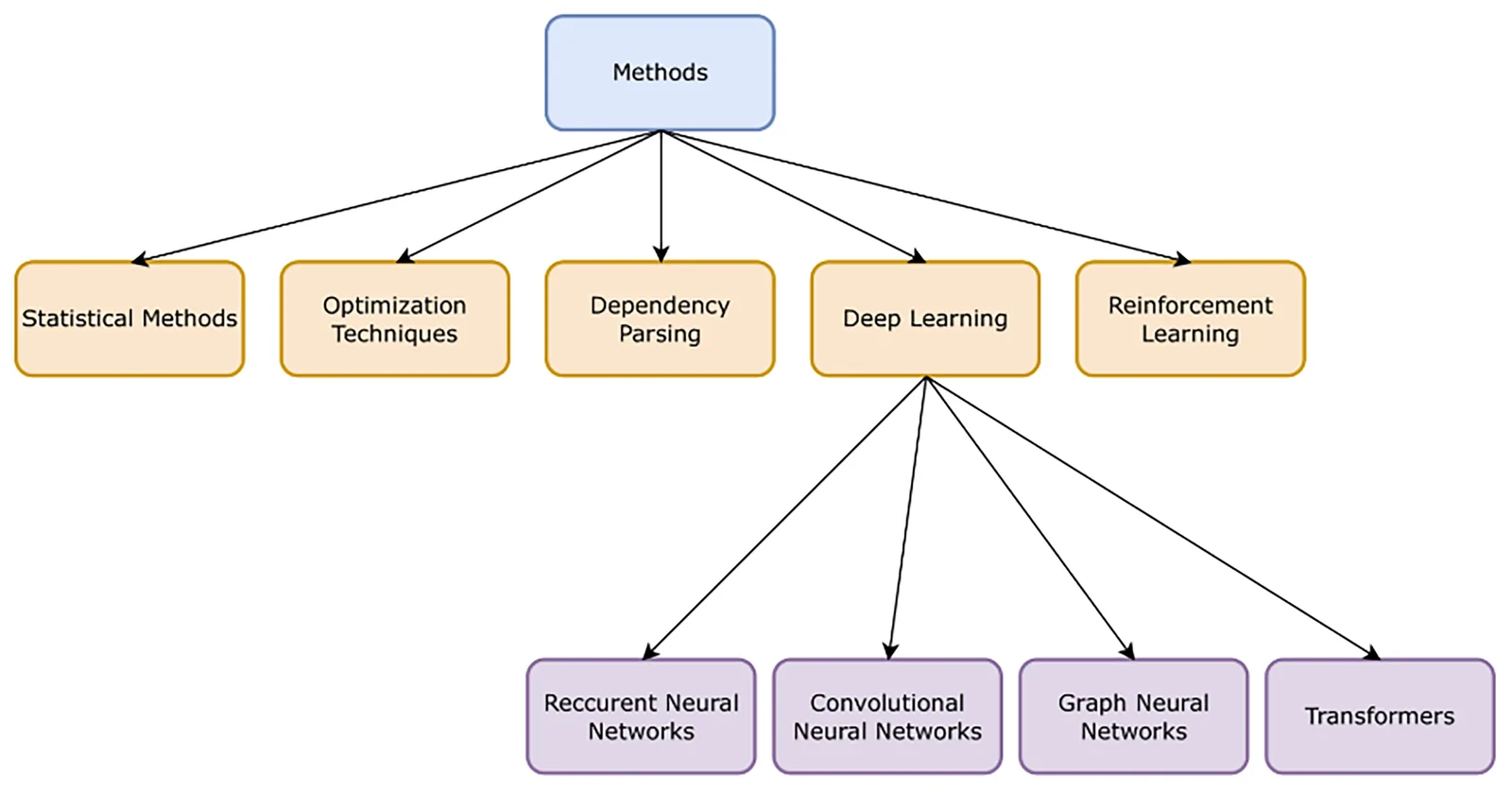
Taxonomy of sentence compression methods [11].

### 1.4. Foundation large language models

Central to LLMs are foundation models, such as ChatGPT and DeepSeek, which provide a powerful framework for creating new GenAI content, offering capabilities for natural language understanding and generation, image processing and more. Broadly, foundation LLMs are categorized as follows:

— **open-source LLMs**: models whose source code, architecture and model weights are publicly available for use, modification and distribution [12]. This openness enables users to develop customized applications with tailored language-generation capabilities. Examples include Meta's LLaMA, DeepSeek R1 and Alibaba's Qwen; and
— **proprietary LLMs**: models developed and owned by specific companies using private training data and infrastructure. They are typically pre-trained on large, diverse datasets and are ready for immediate deployment for general-purpose tasks. Examples include OpenAI's GPT-4, Google Gemini and Anthropic Claude.

In summary, a foundation model is a large, general-purpose AI trained on massive public data, and deploy it for your use-case by writing well-crafted prompts (or light fine-tuning) that steer its tone, structure and reasoning, and then further improve accuracy by adding retrieval-augmented generation (RAG), which fetches the most relevant facts from your own knowledge base at query time and injects them into the prompt, so the model produces up-to-date, grounded answers without needing full retraining. We note that ‘foundation model’ here follows the Stanford definition (broad pre-training plus adaptability, essentially by fiat); a richer view places such models on a neuro-symbolic spectrum, from purely neural to systems with explicit symbolic reasoning.

In table 2, we show examples of foundation models and tools available for LLM programming.

**Table 2.** LLMs, foundational models and LLM tools.

LLM category	traditional LLMs	multi-modal LLMs	large ‘deep’ models	domain-specific LLMs	optimizing LLM tooling /techniques	underlying network /interpretability
models/tools	ChatGPT-4o (GPT-4-turbo), DeepSeek V3, GPT-3.5-turbo, LLaMA-2 (70B), Claude 2	GPT-4o Vision, Gemini 1.5 Pro, DeepSeek-VL2, DALLE-3 (image), Stable Diffusion XL, LLaVA 1.5, AutoGen/AutoGPT	GPT-4, Claude 3 Opus, Gemini 1.5 Pro (1 M-token context), DeepSeek R1, SONAR	BloombergGPT (finance), BioGPT (biomedical), Med-PaLM 2 (medical), FinGPT, Code Llama/StarCoder (software), Galactica (science)	DeepSpeed-ZeRO/MoE, LoRA and QLoRA adapters, FlashAttention-2, BitsAndBytes 8-/4-bit quantization, vLLM and ONNX Runtime/TensorRT-LLM	GPT-2, BERT, TransformerLens, Captum, Ecco, Attention-Roll-out (Acme interp suite)

A major concern for users of foundation LLMs is data privacy and security. Namely, foundation models ‘harvest’ organizations’ private and sensitive data during training. The key application areas include sensitive healthcare data, which is heavily regulated and highly valuable financial data. LLMs often ingest vast and diverse datasets, increasing the likelihood of unintentionally memorizing and reproducing personally identifiable information [13,14].

LLMs are subjected to passive privacy leakage and active privacy attacks [15,16]. Based on the location of privacy protection, LLMs can be grouped into three categories: (i) privacy protection in pre-training, (ii) privacy protection in fine-tuning, and (iii) privacy protection in inference.

Broadly, there are three privacy strategies:

— **foundation models**: configure privacy settings to use only sensitive data for prompts and not as part of LLM training. Foundation models now provide comprehensive settings to address privacy concerns during pre-training and fine-tuning;
— **privacy techniques:** use sophisticated privacy techniques, such as differential privacy, data cleaning and federated learning, to mitigate privacy risks; and
— **custom LLM**: install and customize a ‘local’ LLM either by purchasing a pre-trained foundational LLM or installing an open-source LLM, which may require training.

## 2. Large language model taxonomy

This section introduces our LLM taxonomy and lists prominent examples, with details and references provided in the sections below:

— **traditional LLMs**: basic LLMs are trained on large datasets using deep learning techniques. LLM prompt *engineering* techniques, such as chain-of-thought (CoT) and RAG, can be used to optimize traditional LLM performance;
— *small language models* (*SLMs*): SLMs are smaller in scale and scope, trained on domain-specific data, and designed to be more computationally efficient, cost-effective and suitable for specific tasks or domains;
— *LLMs*: LLMs are AI models that are trained on vast amounts of data and context and excel in processing, understanding and generating human language, and use deep learning techniques, specifically transformer models, to learn relationships;
— *prompt engineering* (*PE*) is the process of designing and refining GenAI prompts, which are basically instructions, questions or guidelines that guide LLM responses. An example is *CoT*;
— **multi-model LLMs:** composite models have been proposed in which multiple specialized models (experts) work together:
— *mixture of experts* (*MoE*)*:* MoE is a machine learning technique in which multiple specialized models (experts) work together, with a gating network selecting the best expert for each input;
— *multimodal LLMs (MLLMs)*: MLLMs are AI systems that also understand and generate various types of data. However, unlike traditional LLMs that primarily focus on text, MLLMs expand this capability by integrating multiple modalities, enabling them to perform more complex and versatile tasks;
— *agentic AI*: it is mostly an LLM system composed of ‘agents’ that can make decisions and act autonomously to achieve specific goals;
— **large deep models**: what we refer to as ‘deep’ models use NLP combined with reasoning capabilities and apply structured reasoning techniques;
— *large reasoning models* (*LRM*): LRM use NLP combined with reasoning capabilities and are trained to apply structured reasoning techniques when responding to prompts;
— *large action models* (*LAMs*): LAMs go beyond generating text, such as an LLM, by understanding and executing actions based on user input and context;
— *large context/contextual* models: large context models and contextual models address different aspects of the context;
— **domain-specific LLMs:** LLMs and transformer models are tailored for specific domains, comprising (i) specific data types, such as numeric or images; (ii) an industry sector, such as finance, law, medicine or education and (iii) multiple domains:
— *large quantitative models* (*LQM*): LQM is specifically used to process, analyse and generate quantitative data rather than natural language;
— *vibe coding LLMs:* an approach to programming and application development that frequently relies on LLMs for code generation, refinement and debugging;
— *financial LLMs:* finance/quant models specialized in processing financial data, market analysis and quantitative reasoning;
— *legal models:* legal models are designed to understand, interpret and generate legal language, documents and case law;
— **optimizing LLMs** – incorporating specialist mechanisms to optimize an LLM, falling between prompt engineering and network interpretability. The three main reasons for this are efficiency, accuracy and privacy;
— *quantization LLMs:* quantization reduces model size by storing weights with lower-*precision* formats, such as INT8, rather than FP32 or FP16;
— *privacy-preserving LLMs:* privacy-preserving/differentially privacy LLMs incorporate mechanisms that limit the ability to memorize or leak sensitive training data;
— **underlying network interpretability:** understanding the internal reasoning processes of trained neural networks. Several methods have been developed to improve the interpretability of transformers, focusing on techniques such as attention visualization, attribution methods and modifications of the model architecture itself;
— *mechanistic interpretability* (*MI*) seeks to understand the internal reasoning processes of trained neural networks and gain insight into how and why they produce outputs; and
— representation *engineering* (*RepE*)*:* RepE, or activation engineering, seeks to understand and control AI by observing and manipulating internal representations—weights or activations—that AI uses to understand and process information.

## 3. Traditional large language models

Traditional LLMs are broadly divided into basic (unoptimized) and optimized models using prompt engineering. As is well known, prompt engineering involves crafting clear, concise prompts that direct GenAI systems towards the desired outputs. By carefully selecting keywords, contextual cues or domain-specific languages, developers can influence how models interpret queries and shape their final responses [17].

**Traditional LLMs**: basic LLMs are trained on large datasets and use deep learning techniques. LLM prompt engineering techniques, such as CoT and RAG, can be used to optimize traditional LLM performance [18]:

— *SLMs*: SLMs are smaller in scale and scope (e.g. TinyZero), trained on domain-specific data, and designed to be more computationally efficient, cost-effective and suitable for specific tasks or domains [19];
— *LLMs*: LLMs are AI models that are trained on vast amounts of data and context and excel in processing, understanding and generating human language, and use deep learning techniques, specifically transformer models, to learn relationships;
— *PE* is the process of designing and refining GenAI prompts, which are basically instructions, questions or guidelines that guide LLM responses. An example is CoT reasoning;
— *CoT:* CoT, tree of thought, graph of thought, forest of thought models, etc. extend standard LLMs by explicitly planning sequences of intermediate reasoning steps or actions to achieve specific goals. These models enhance traditional CoT reasoning by exploring multiple possible reasoning paths, structured as trees or graphs. This approach allows for the systematic exploration of alternatives and backtracking, improving problem-solving capabilities in complex, multistep tasks that require strategic planning and evaluation [20]; and
— *RAG*: RAG models enhance traditional LLMs by integrating specialist knowledge-retrieval systems. Instead of relying solely on pretrained knowledge, these models dynamically access relevant documents or databases during inference, improving the accuracy and grounding outputs in up-to-date or domain-specific information. Enhances general LLMs with external knowledge access [21,22].

### 3.1. Small language models

SLMs are smaller in scale and scope, trained on domain-specific data and designed to be more computationally efficient, cost-effective and suitable for specific tasks or domains.

The key differences between SLMs and LLMs [19] are: (i) *size and parameters*—SLMs have a smaller number of variables, typically ranging from a million to a billion rather than a trillion; (ii) *computational requirements*—SLMs require less computational power and memory, hence faster to train and deploy; (iii) *training data*—SLMs are trained on smaller, more focused datasets, allowing for specialization in specific tasks; and (iv) *performance*—SLMs can perform well on specific, well-defined tasks. Parameter count alone is a weak criterion (what counts as a parameter varies, e.g. dense versus sparsely-activated experts); the small/large distinction is better read across several qualitative axes—compute and data scale, breadth of competence and deployment footprint.

SLMs are well suited for scenarios where resources are limited, such as on-device AI, edge computing and mobile applications.

### 3.2. Large language models

LLMs, as discussed, are trained with vast amounts of data and context and excel at processing, understanding and generating human language. Deep learning techniques, specifically transformer models, are used to learn these relationships.

Basic LLMs were largely unoptimized, with current LLMs built using foundation models optimized using prompt engineering.

### 3.3. Prompt engineering

PE is the process of designing and refining GenAI prompts—basic instructions, questions or guidelines—that guide LLM responses. Prompts are best understood as program-like guidance to a capable but unreliable generator: their structure (role, instructions, constraints and exemplars) changes behaviour; for example, adding ‘and do not fabricate any references’ to a request for legal citations can materially change the output.

LLM optimization is the process of refining and enhancing the performance and efficiency of LLMs. This includes improving the computational efficiency, text generation accuracy and handling of biases, and even reducing the environmental impact of training and deploying these models.

Prominent optimization technologies include:

— *zero/few shot learning*: zero-shot learning involves a model that recognizes new categories or classes that are not observed during training. On the other hand, few-shot learning allows a model to learn from a small number of new class examples [17];
— *CoT:* CoT prompting simulates a form of human-like reasoning by encouraging LLMs to break down complex tasks into logical steps. Instead of jumping directly to a final answer, the model is nudged to articulate intermediate reasoning, thereby improving the interpretability and quality of its output [23];
— *prompt frameworks* are structured methodologies for building prompts that ensure the consistency, safety and high performance of LLM systems; and
— *RAG*: RAG combines LLM outputs with information retrieved from trusted external sources. LLM first generates a response or interpretation of a query, after which a retrieval component sources additional data or facts to refine the output. This hybrid approach mitigates the limitations of purely generative models by grounding responses in verifiable evidence, thereby reducing errors and hallucinations [24]. RAG also partly compensates for the absence of continual learning, injecting current facts at inference rather than updating the model's weights.

#### 3.3.1. Properties

Table 3 summarizes the application properties of LLMs: SLMs, classic (unoptimized) LLMs and LLMs optimized using techniques such as PE and RAG.

**Table 3.** Traditional LLMs’ properties.

model type	properties (typical characteristics)	applications (use cases)	strengths
SLMs	smaller scale, domain-focused datasets; specialized tasks; low parameter count (millions)	on-device AI, edge computing (e.g. mobile apps, Internet of Things); specific domain tasks	low computational requirements (faster and lightweight); cost-effective for narrow tasks
LLMs	general-purpose models trained on trillions of tokens; very high parameter count (billions–trillions)	general-purpose NLP applications across domains (chatbots, translation, summarization, etc.)	high versatility and state-of-the-art performance; can serve as foundation models adaptable to many tasks
optimized LLMs	general-purpose LLMs enhanced with specialized techniques (prompt engineering, fine-tuning, retrieval, etc.)	all tasks handled by base LLMs, but with improved accuracy, relevance or efficiency (e.g. complex reasoning with CoT, up-to-date info with RAG)	enhanced performance on complex or specialized tasks (through CoT, RAG, etc.); often avoids the need for model retraining by leveraging prompt or plugin strategies

## 4. Multi-model large language models

While a single (traditional) LLM relies on one comprehensive model for a range of tasks, multi-model LLMs rely on the collaboration of several specialized models. The multi-model approach is intended to achieve a greater level of accuracy for a set of specific tasks. Examples in this category include a combination of experts, MLLMs and agentic AI. (We use ‘multi-model’ for systems combining several cooperating models and ‘multimodal’ for a single model spanning several data modalities; these are distinct axes.)

Multi-model LLMs are composite models in which multiple specialized models (experts) work together:

— *MoE:* MoE is a machine-learning technique in which multiple specialized models (experts) work together, with a gating network selecting the best expert for each input [25];
— *MLLMs:* MLLMs are AI systems that understand and generate various types of data. However, unlike traditional LLMs that primarily focus on text, MLLMs expand this capability by integrating multiple modalities, enabling them to perform more complex and versatile tasks [26]; and
— *agentic AI:* it is mostly an LLM system composed of ‘agents’ that can make decisions and act autonomously to achieve specific goals. Unlike traditional AI systems, AI agents are designed to pursue complex goals and execute tasks with minimal human supervision. They act on complex contexts, iterate towards goals through an external control loop and tool calls, rather than through any capacity for comprehension [27].

### 4.1. Mixture of experts

The MoE is an LLM technique in which multiple experts work together, with a gating network selecting the best expert for each input. These ‘experts’ are individual neural networks trained to specialize in different aspects of the task, such as specific linguistic patterns, syntactic structures or semantic understanding. A ‘gating network’ acts as a router, deciding which experts to activate for a given input. The gating network uses a learned gating function (e.g. a neural network) to assign probabilities to each expert, selecting the top *k* most likely experts. In MoE models, ‘sparsity’ is a key characteristic because only a subset of experts is active for any given input. This implies that MoE models can handle a large number of parameters that are computationally efficient during inference.

MoE works as follows [25]: (i) *input*—an input sentence or paragraph is fed into the MoE model; (ii) *routing*—the gating network analyses the input and assigns a weight to each ‘expert’ based on its relevance; (iii) *activation*—the ‘experts’ with the highest weights are activated, with their sub-networks processing the input; and (iv) *output*—the outputs from the activated experts are combined (often using a weighted average based on the gating network weights) to produce the final output of the MoE model.

### 4.2. Multimodal large language models

MLLMs are AI systems that understand and generate various types of data, but unlike traditional LLMs that primarily focus on text, MLLMs expand this capability of traditional LLMs by integrating multiple modalities, enabling them to perform more complex and versatile tasks. This means that MLLMs can interpret a variety of data types, including: (i) *sensory data*—information from motion sensors; (ii) *GPS data*—information from GPS and other tracking devices; (iii) *three-dimensional data*—spatial data and three-dimensional representations; (iv) *structured data*—information from spreadsheets or databases requiring numerical or categorical analysis; and (v) *mixed documents*, such as webpages or multimedia documents that combine text, code, images, etc.

### 4.3. Agentic artifical intelligence

Agentic AI are LLMs composed of ‘agents’ that can make decisions and act autonomously to achieve specific goals. Unlike traditional LLM systems, these agents execute tasks independently and are designed to pursue complex goals and execute tasks with minimal human supervision. They act on complex contexts, iterate towards goals through an external control loop and tool calls, rather than through any capacity for comprehension.

The key agentic AI concepts [27] are: (i) *autonomy—*agentic AI systems are capable of operating independently and making decisions without constant human guidance; (ii) *goal-oriented—*they are designed to pursue specific objectives and can adapt their actions to achieve those goals; (iii) *reasoning/planning—*agentic AI uses sophisticated reasoning and iterative planning to solve complex, multistep problems; (iv) *adaptability—*systems learn from their experiences and adjust their behaviour based on feedback and changing conditions; and (v) *integration—*agentic AI often combines different AI techniques, such as LLMs, ML and reinforcement learning.

#### 4.3.1. Properties

In table 4, we attempt to summarize the properties of multi-model LLMs.

**Table 4.** Multi-model LLM properties.

model type	properties (typical characteristics)	applications (use cases)	strengths
MoE	route each input token to a sparse subset of specialized ‘expert’ sub-networks chosen by a learned gating router	— sparse activation (only approx. 1–2 experts per token) — gating network for dynamic routing — parameters scale ≫ compute cost	massive parameter counts with reasonable inference cost; lets you train domain-specialized experts in one model
MLLMs	fuse textual and non-textual information (images, audio, video, sensor, code, etc.) in a single model that understands and generates across modalities	— modality-specific encoders feeding a shared transformer — cross-attention for aligning modalities — large unified embedding space	creative and analytical tasks needing joint reasoning: visual Q&A, data-rich chatbots and robo-control
agentic AI	compose one or more LLMs into autonomous agents able to plan, tool-call and iterate towards a goal with minimal human oversight	— planner → executor loops — memory / scratch-pads — tool-calling / code-execution — often combines RAG + reinforcement learning	long-horizon tasks: multistep research, coding, workflow automation, and test-case generation

## 5. Large deep models

We introduce the term large ‘deep’ models for LLMs that use NLP combined with specialist capabilities and are trained to apply structured techniques when responding to prompts:

— *LRM*: LRMs use NLP combined with reasoning capabilities and are trained to apply structured reasoning techniques when responding to prompts [28];
— *LAM*: LAMs go beyond generating text, such as an LLM, by understanding and executing actions based on user input and context [29];
— *large context* models: large context models are a specific type of LLM designed to handle significantly larger amounts of text within their processing window (context window) [30,31]; and
— *large concept models* (*LCM*): LCM language models process natural language at the concept level rather than analysing individual words [32].

### 5.1. Large reasoning models

LRMs use NLP combined with reasoning capabilities and are trained to apply structured reasoning techniques when responding to prompts. LRMs are trained differently from LLMs [28]. They use: (i) *enriched training data*—not just language patterns but also examples designed to teach reasoning, such as real-world scenarios with clear outcomes; (ii) *reinforcement learning*—used to reward correct or logically consistent answers and penalize incorrect ones; (iii) *human feedback*—reinforcement learning combined with human feedback (RLHF) to guide and refine the model's outputs, helping it learn nuanced reasoning strategies that align with domain expertise; and (iv) *prompt engineering*—CoT deployed to guide the model through multistep tasks or layered questions.

#### 5.1.1. Large reasoning model types of reasoning

The four main types of ‘reasoning’ commonly used in LRMs are set out below; note that any prediction from a learned model is itself inductive, so these labels describe the pattern imitated, not guaranteed soundness: (i) deductive reasoning—applying general rules to a specific case so that, if the premises hold, the conclusion follows, requiring strict adherence to established rules or facts; (ii) *inductive reasoning—*draws general conclusions from specific observations in training data, supporting probabilistic predictions rather than guaranteed outcomes, (iii) *abductive reasoning—*involves inferring most likely explanation based on incomplete or uncertain data; and (iv) *analogical reasoning—*involves identifying similarities between different situations or datasets and applying insights from one context to another, to recognize relational patterns across examples and transfer learning.

### 5.2. Large action models

LAMs are a type of LLM system that go beyond generating text by understanding and executing actions based on user input and context. Think of it as a ‘can-do’ cousin to the ‘talk-only’ LLM. LAMs can interact with various systems and interfaces to perform tasks, such as making reservations, booking appointments or completing forms.

The key characteristics of LAMs [29] are: (i) *action-oriented—*LAMs are designed to perform actions in software or physical environments rather than process text; (ii) *understanding intent—*LAMs analyse user input (e.g. text, voice, etc.) and interpret the underlying goal or intention behind the request; (iii) *task decomposition—*breaks down complex tasks into sub-tasks; (iv) *execution—*LAMs execute actions by interacting with other systems, applications or physical devices; (v) *adaptability—*LAMs adapt their actions based on feedback, learning and improving their performance over time; (vi) *LLM integration—*LAMs build upon traditional LLMs, leveraging their capabilities, adding action-oriented components; and (vii) *hybrid approach*—LAMS can combine neural networks with symbolic reasoning or planning algorithms to enhance understanding of context and execute tasks effectively.

### 5.3. Large context/contextual models

Large context models and related large contextual models address different aspects of context: (i) *context models* provide LLMs with more information to process at once, improving their ability to understand and respond to complex queries; (ii) *contextual models* focus on how information is related within a specific context to retrieve relevant information from a larger (RAG) knowledge base [30,31].

The evolution of context windows in LLMs represents one of the most significant advances in terms of AI capability and utility. The context window (or ‘context length’) of an LLM is the amount of text, in tokens, that the model can consider or ‘remember’ at any one time. A larger context window enables an AI model to process longer inputs and incorporate more information into each output.

### 5.4. Large concept models

Meta's LCMs are models that process language at the concept level, analysing larger sections of text to extract underlying ideas, rather than analysing individual words. Instead of relying on language-specific patterns, LCMs store meaning, making them adaptable to tasks such as multilingual summarization, translation and cross-format content generation. They perform autoregressive sentence prediction and are built on the SONAR embedding space, which allows them to process text in over 200 languages and speech in 76 [32].

LCMs follow a three-step pipeline:

— *concept encoder* (*SONAR*) input (text or speech) is broken into sentences in a semantic embedding space. Each sentence is converted into a semantic ‘concept embedding’ space using a tool called SONAR (which works in 200 languages and supports text and speech);
— *LCM core* performs reasoning and prediction using a transformer model, which causes the concept sequence to generate the output concepts. Concept embeddings are processed by the LCM to perform tasks such as summarizing or translating; and
— *concept decoder* (*SONAR*) translates the model output (embeddings) back into a human-readable natural language (or speech) output.

The key characteristics of LCMs [33]: (i) *multi-format—*multilingual and multiformat flexibility, working in 200+ languages and supporting text and speech, without extra training; (ii) *generalization—*generalizing to new tasks by using a language-independent system, allowing it to handle new languages and tasks without extra training; (iii) *coherence—*processing full sentences, keeping responses clearer and more structured over long texts; and (iv) *efficiency*—efficient in handling context by using compact sentence representations, making it easier to process long documents efficiently.

#### 5.4.1. Properties

In table 5, we again summarize the properties of large deep models.

**Table 5.** Large deep models.

model type	properties (typical characteristics)	representative examples	trade-offs
LRM	train the LLM to explicitly plan, justify or prove its answers rather than produce fluent text only	OpenAI o1/o3, DeepSeek-R1, Google Gemini ‘thinking’ variants and DeepMind AlphaGeometry	cost of multi-turn deliberation, reward hacking (spurious chains) and difficulty of automatic grading of reasoning quality
LAM	couple an LLM with action execution layers so the model can read intent → plan → act inside software or the physical world	Devin (‘AI software engineer’), Adept Fuyu-8B, OpenAI GPT-4o + Actions, AutoGPT/CrewAI agent frameworks and robotics model PaLM-E	safety and permissioning, grounding to real-world state, cascading errors from misplanned steps and latency/cost of tool chains
large context / contextual models	scale the effective working memory of the model by (i) vastly enlarging the native context window and/or (ii) tightly integrating RAG	Anthropic Claude 3 Opus (200K ctx), Google Gemini 1.5 Pro, Microsoft phi-3-vision-128k, wrapper libs LlamaIndex and Haystack	quadratic attention cost, long-range forgetting, citation faithfulness, retrieval noise and recency drift
LCM	move from word-level tokens to sentence/idea-level ‘concept embeddings’ that are language-agnostic and multimodal	Meta LCM on the SONAR embedding space (text + speech, 200+ languages)	coarse granularity may miss nuance, aligning concept spaces across modalities and limited public training data

## 6. Domain-specific large language models

Domain-specific LLMs are the most rapidly developing category of LLMs. These and related transformer models are tailored for specific domains, comprising (i) specific data types, such as numeric or video; (ii) an industry sector, such as finance, law, medicine or education; and (iii) multiple domain models:

— *LQM*: LQM is specifically used to process, analyse and generate quantitative data rather than natural language [34];
— *vibe coding LLMs*: an approach to programming and application development that relies heavily on LLMs for code generation, refinement and debugging [35];
— *financial LLMs*: finance/Quant models specialized in processing financial data, market analysis and quantitative reasoning. They are trained on vast datasets from financial markets, news and economic reports, enabling applications such as trading strategies, risk management, portfolio optimization and financial forecasting (Bloomberg GPT, FinGPT and LQMs) [36];
— *legal models:* legal models are designed to understand, interpret and generate legal language, documents and case law. By fine-tuning the legal corpora, these models assist with contract analysis, legal research, compliance monitoring and automated drafting, improving efficiency and reducing errors in legal workflows (e.g. Harvey and CaseLawBERT) [37]; and
— *educational LLMs*: educational/pedagogical models are fine-tuned to provide personalized tutoring, explanations and assessments. They adapt to learners’ skill levels, offer interactive feedback, support diverse subjects and enhance their learning experiences through AI-driven teaching assistance [38].

In addition, we can also include these specialist LLMs:

— *biochemical LLMs*: bio/chem models are trained on biochemical or molecular data rather than natural language. They predict molecular properties, protein structures or chemical reactions. Such models are now pivotal in drug discovery, genomics and materials science, where textual LLMs are insufficient for structured scientific data (e.g. MolBERT and AlphaFold) [39];
— *scientific LLMs*: scientific/research models are tailored for scientific and academic domains, trained on research papers, technical articles and domain-specific terminology. They support literature summarization, hypothesis generation and data extraction, thereby accelerating research discovery and knowledge dissemination. (e.g. Galactica and SciBERT) [40]; and
— *long-document LLMs*: long-document models specialize in processing extensive texts, such as books, research papers, contracts or technical documentation. These models incorporate architectural changes (e.g. sparse attention or memory modules) to handle long-range dependencies and preserve coherence across thousands of tokens. Examples include BigBird, Longformer, Hierarchical Attention Transformers (HATs) and BlockBERT [41].

### 6.1. Large quantitative models

LQMs are designed specifically to process, analyse and generate quantitative data rather than natural language. Unlike traditional LLMs, which excel in language-related tasks, LQMs focus on handling large-scale numerical datasets and performing complex calculations, statistical analysis, predictive modelling and optimization tasks. These models are envisioned as quantitative counterparts of traditional LLMs, providing data-driven insights and solutions to numerical problems across various industries.

LQMs use deep learning frameworks for time-series data with convolutional neural networks (CNNs), recurrent neural networks (RNNs) and long short-term memory (LSTM) networks, combined with custom layers for advanced statistical models using Bayesian networks or regression models.

The key characteristics of LQMs [34] are: (i) *reason style—*deterministic or stochastic mathematical reasoning; (ii) *interpretability*—typically more interpretable, especially in structured scientific models; (iii) *architecture—*mathematical or physics-based models, often combined with ML techniques; (iv) *training*—scientific theories, simulations and specialized datasets; (v) *real-time usage—*running simulations, forecasting and optimizing systems; and (vi) *error handling*—with LQMs, errors tend to be mathematical or computational, often easier to debug.

### 6.2. Vibe coding large language models

Vibe coding frequently involves using LLMs to generate, refine and debug code, with developers focusing on the overall ‘vibe’ or direction of the project rather than crafting every line of code. In ‘vibe coding,’ developers focus on describing the desired functionality in natural language (‘the vibe’), rather than writing exact code, and the LLM generates and iteratively refines the code. This contrasts with traditional PE for code (which might require very precise prompts); vibe coding is more about guiding the AI's outputs at a high level and letting the model fill in details. We can also mention common application areas such as website development, where startups like Lovable (https://lovable.dev/) are at the forefront.

The key characteristics of vibe coding LLMs [35]: (i) *natural language—*vibe coding describes their desired functionality in human language; (ii) *refinement—*the developer reviews and refines the generated code iteratively, providing feedback in natural language; (iii) *guidance—*focus on the desired ‘creative’ outcome rather than granular code details; (iv) *prototyping—*significantly speed up the development of early-stage prototypes; and (v) *accessibility—*make software development more accessible to individuals without extensive programming and technical backgrounds. An interesting development of vibe coding is the Darwin Gödel Machine [42], a self-improving system that iteratively modifies its own code.

### 6.3. Legal large language models

Legal services and the judiciary are being ‘revolutionized’ by AI legal assistants covering contract generation, legal advice, online dispute resolution and internet courts [43]. Legal technology (i.e. LegalTech for lawyers, LawTech for clients and JudicialTech for the judiciary) encompasses software and technology solutions that support and enhance legal services. It aims to improve the efficiency, accuracy and accessibility within the legal industry by automating tasks, streamlining processes and providing new ways to deliver legal services. Legal technology can include everything from basic tools, such as e-signature platforms, to more advanced applications, such as AI-powered legal research and contract analysis [37].

The key characteristics of legal technology and LLM assistants [37] are as follows: (i) *automation—*legal technology can automate repetitive tasks, such as document drafting, data entry and legal research, freeing up legal professionals to focus on more complex tasks; (ii) *LegalTech software—*involves specialized software and online platforms for legal professionals that help with tasks like document management, legal research, contract generation/analysis and client communication; (iii) *LawTech software*—client (LLM) software providing legal advice, draft contracts, case routing and estimation of litigation success. Making legal services more accessible to individuals and businesses; and (iv) *JudicialTech software*—judicial platforms for judicial stakeholders, so-called smart/internet courts and online dispute resolution and LLM assistants for judges and court officials.

By leveraging legal technology, legal professionals, clients and the judiciary can improve the speed and accuracy of their work, potentially reducing costs and errors, and improving access to justice.

### 6.4. Educational large language models

Education is probably the most active sector for LLM assistants [38].

Figure 2 illustrates LLM educational activities divided into pedagogical agents and domain-specific agents [44].

**Figure 2.**
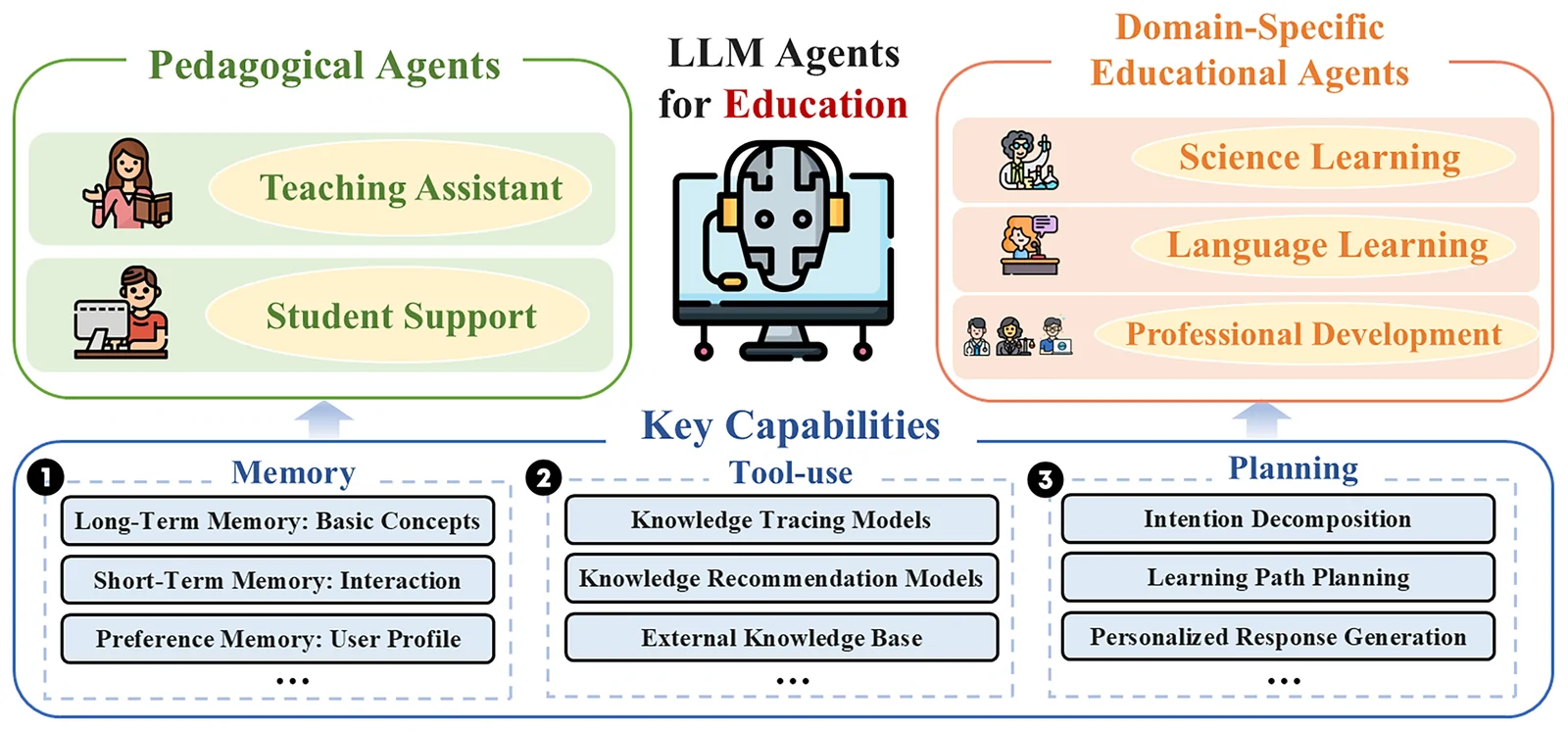
LLM pedagogical agents for education [44].

The benefits of LLMs in education for teachers and students include (i) *personalization—*personalized learning content; (ii) *automation—*automating tasks such as lesson planning and grading; (iii) *feedback*—providing quick help and feedback to students; and (iv) *innovation—*cutting-edge learning resources and technology, such as interactive simulations and games.

#### 6.4.1. Properties

Table 6 summarizes the properties of the domain-specific LLMs.

**Table 6.** Domain-specific models.

model type	properties (typical characteristics)	applications (use cases)	strengths
LQM	treat numerical / time-series input as first-class data and perform deterministic or stochastic mathematical reasoning	CNN/RNN/LSTM blocks, Bayesian layers and physics-informed modules	forecasting, simulations, optimization and ops research
vibe-coding LLMs	developers describe the vibe in NL; the model generates, refines and debugs the code iteratively	natural language-to-code generation loops and RLHF with code quality rewards	ease and speed of development
financial LLMs	fine-tuned on market data, news and filings for quantitative reasoning over finance	domain RAG, numerical adapters and long-context windows	trading signals, risk management, forecasting and research Q&A
legal LLMs	understand statutes, contracts and case law; draft and analyse legal text	long-doc attention, citation retrieval and jurisdiction filters	contract review, legal research and compliance checks
educational LLMs	personalized tutoring and teacher co-pilots that adapt to learner level	student-profile memory and interactive feedback loops	tailored explanations, automated grading and lesson generation

## 7. Optimizing large language models

We introduce the term ‘optimizing LLMs’ for models incorporating a specific (i.e. non-prompting) mechanism to optimize an LLM: because ‘optimize’ requires an objective, we state it: here the objective is inference efficiency, privacy or robustness, as distinct from training-loss, alignment or task-utility optimization:

— *quantization LLMs*: quantization reduces model size by storing weights in lower-precision formats such as INT8 rather than FP32 or FP16 [45];
— *privacy-preserving LLMs*: privacy-preserving/differentially privacy LLMs incorporate mechanisms that limit the ability to memorize or leak sensitive training data. Differential privacy ensures that no individual data point has a measurable influence on the model's outputs, protecting user confidentiality and enabling compliance with data protection regulations, such as general data protection regulation and Health Insurance Portability and Accountability Act [46];
— *adversarial-robust LLMs*: these models are trained or adapted to resist adversarial prompts and malicious input manipulation such as prompt injection or jailbreak attacks. These techniques include robust training, defensive fine-tuning and input sanitization. Such models are essential for deploying LLMs in security-critical environments, including legal, military and enterprise settings [47]; and
— *thinking LLMs*: aimed at improving general instruction following in LLMs by explicitly incorporating internal ‘thought’ processes before generating responses [48].

Again, these optimizing LLMs might be included:

— *alignment-tuned models*: alignment-tuned (RLHF and constitutional AI) models are fine-tuned to align more closely with human values, ethical principles or domain-specific guidelines. RLHF and constitutional AI approaches help ensure that the model's output remains safe, helpful and contextually appropriate, particularly in sensitive or high-stakes applications [49];
— *instruction-tuned LLMs*: instruction-tuned/role-conditioned LLMs are further fine-tuned to follow explicit human instructions or behave according to defined roles (e.g. financial advisor and legal analyst). Instruction tuning improves the alignment of model outputs with user intent, thereby enhancing reliability, safety and usability in real-world scenarios; and
— *tool-using LLMs (function calling and agents)*: these models extend traditional LLM capabilities by invoking external tools, application programming interfaces or functions during generation. By dynamically calling specialized functions or software components, tool-using LLMs can perform tasks beyond pure language generation, such as calculations, database queries and code execution, enabling more practical and accurate outputs [50].

### 7.1. Quantization of large language models

Quantization is a technique used within LLMs to convert the weights and activation values of high-precision data, usually 32-bit floating point (FP32) or 16-bit floating point (FP16), to lower-precision data, such as an 8-bit integer (INT8). In essence, the quantization process involves mapping weights stored in high-precision values to lower-precision data types. For LLMs, weights often have distributions centred around zero, making symmetric quantization a common choice. However, activations, particularly after ReLU or GeLU functions, can be strictly non-negative or have highly asymmetric distributions.

The two types of LLM quantization are: (i) *post-training quantization* (*PTQ*), which refers to techniques that quantize an LLM after it has already been trained; and (ii) *quantization-aware training* (*QAT*), which refers to methods of fine-tuning data with quantization in mind.

The key characteristics of quantized LLMs are [51]: (i) *range mapping*—the mapping of weights stored in high-precision values to lower-precision data types; (ii) *granularity*—defines how broadly or narrowly we apply quantization parameters such as scale and zero point across a model; and (iii) *calibration*—involves running inference on a representative dataset to optimize the quantization parameters and minimize the quantization error.

Quantization is an integral part of the LLM landscape. By compressing the size of language models, quantization techniques (e.g. QLoRA, PRILoRA, GPTQ, GGML/GGUF and AWQ) help to increase the adoption of LLMs [51].

### 7.2. Privacy-preserving large language models

LLMs often require extensive datasets for pretraining and fine-tuning, which may include sensitive, proprietary or personally identifiable information. These datasets, coupled with the ability of the models to memorize and reproduce training data, expose risks such as data leakage, membership inference and model-inversion attacks [46].

To address these challenges, researchers have developed various privacy-preserving mechanisms tailored to LLMs. Techniques such as differential privacy, federated learning, cryptographic protocols and trusted execution environments. Differential privacy is the extent to which data must be protected, altered or anonymized to avoid reidentification of private data. Federated learning trains algorithms locally without explicitly changing data. Cryptographic protocols use encryption and digital signatures to ensure secure communication and data exchanges. Trusted execution environments are secure and isolated areas within a device processor that protect sensitive data and code. These approaches are particularly crucial in privacy-sensitive domains, including healthcare and finance, in which breaches of data confidentiality can have severe consequences.

### 7.3. Adversarial-robust large language models

Continuous adversarial attacks have recently demonstrated higher success rates and significantly shorter computation times than their discrete counterparts in LLMs. Adversarial training, which involves online augmentation of the training data of a neural network with adversarial attacks, has consistently been proven to enhance robustness against adversaries [47,52].

#### 7.3.1. Properties

Table 7 summarizes the properties of what we refer to as optimizing LLMs, which provides optimization mechanisms beyond PE.

**Table 7.** Optimizing LLM properties.

approach	properties (typical characteristics)	applications	strengths
quantization LLMs	compress the model by storing weights/activations in lower-precision formats (INT8, INT4) instead of FP32/FP16	PTQ, QAT, per-channel scales and zero-point calibration	running large models on laptops, edge devices or single GPUs; cost-effective serving
privacy-preserving LLMs	inject differential-privacy noise or secure computation, so no single datum measurably affects outputs	differentially private-stochastic gradient descent training, clipping and noise addition and secure aggregation	healthcare, finance, any setting with sensitive data and compliance needs
adversarial-robust LLMs	harden models against jailbreaks, prompt injection and other adversarial inputs	robust/adversarial fine-tuning, input sanitization and rule-based guards	safety-critical, legal or military chatbots; secure RAG pipelines

**Table 8.** Underlying network interpretability.

approach	properties (typical characteristics)	applications	strengths
MI	reverse-engineer an LLM into human-readable features and circuits that explain how specific behaviours arise	activation patching, path-patching, weight lenses and circuit tracing dashboards. TransformerLens toolkit (circuits work by [53,60])	trust-building audits, red-team analysis, safety verification and curriculum for model debugging
RepE	identify linear concept vectors in activation space and add or subtract them at run-time to steer model outputs	concept-vector discovery, similarity scoring and forward-pass vector injection. RepE / activation-addition methods [54]	fine-grained behaviour control, rapid customization without weight updates and content filtering
SAM	train sparse autoencoders on model activations to obtain a set of disentangled, interpretable latent features	encoder–decoder with sparsity constraint and feature selectivity metrics. Sparse-feature autoencoders for LLMs [56]	discovering human-readable features, dimensionality reduction and systematic probing of large models

## 8. Underlying network interpretability

Whereas prompt engineering optimizes ‘prompts,’ newer techniques attempt to decode and manipulate the underlying neural network architecture. Examples include MI [53] and RepE [54]. These interpretability techniques focus on understanding the internal reasoning processes of neural networks to reveal how they process information and make predictions. We also highlight the opportunity for these techniques to adjust specific activation functions using control vectors as a method to increase interpretability in LLMs.

In summary, network interpretability addresses the internal reasoning processes of trained neural networks. Several methods have been developed to improve the interpretability of transformers, focusing on techniques such as attention visualization, attribution methods and modifications of the model architecture itself.

— *MI:* seeks to understand the internal reasoning processes of trained neural networks and gain insight into how and why they produce the outputs that they do [53,55].
— *RepE*: RepE, or activation engineering, seeks to understand and control AI by observing and manipulating internal representations—weights or activations—that AI uses to understand and process information [54].
— *Sparse models*: sparse models (e.g. sparse autoencoders and mixture-of-sparse experts) employ techniques that activate only a subset of the network's parameters for each input, reducing computational load and improving efficiency. Sparse autoencoders enforce sparsity constraints during training, while mixture-of-sparse experts dynamically select relevant experts, balancing performance with resource usage (i.e. incorporating a sparsity constraint during training to encourage the network to learn a representation in which only a small number of hidden units are active at any given time) [56].

We also include, for completeness:

— *neurosymbolic models (NeSy)*: NeSy combine neural network learning with symbolic reasoning systems. This hybrid approach integrates the pattern recognition strengths of neural models with the structure and logic of symbolic systems, enabling better reasoning, rule following and interpretability in tasks that require consistency, such as legal reasoning or theorem proving [57].

### 8.1. Mechanistic interpretability

MI [53] focuses on understanding the internal workings of machine-learning models, particularly LLMs, by reverse-engineering their algorithms into human-understandable mechanisms [58]. It aims to go beyond simply observing inputs and outputs, striving to identify the specific components and processes within the underlying neural network that lead to particular behaviours. This approach seeks to reverse-engineer neural networks by analysing the learned weights to uncover human-interpretable algorithms and learned weights to understand how information flows and computations are performed [55]. The goal of MI is to understand the internal workings of a neural network, typically by identifying the circuits within the model responsible for specific behaviours. Additional examples of this approach are provided by [59,60]. MI is critical to improving trustworthiness in LLMs, which remains a critical topic when considering which LLM to use for different use cases, such as privacy and security.

At the heart of MI are two key concepts: ‘features’ and ‘circuits’, pivotal in deciphering the operations of neural networks and understanding how information is processed. The key characteristics of MI are: (i) *features—*refer to the specific properties or patterns that a neural network learns to recognize and process; (ii) *circuits—*are groups of neurons or ‘functional units’ within a neural network that work together to perform specific computations; and (iii) *relationships—*features are the ‘what’—the information being processed—while circuits are the ‘how’—the pathways and computations that process this information.

### 8.2. Representation engineering

RepE, or activation engineering, is a new paradigm for optimizing the behaviour of LLMs. It involves creating vectors of activations that cause desired changes to the output text when added to the forward passes of a frozen LLM. RepE is a set of methods used to understand and control the behaviour of LLM neural networks. This is done by first identifying a linear direction in the activations related to a specific concept [54], a type of behaviour, or a function, which we call the concept vector. During the forward pass, the similarity of activations to the concept vector can help detect the presence or absence of the concept direction. Furthermore, the concept vector can be added to the activations during the forward pass to steer the behaviour of the LLM towards the concept direction.

The key representation characteristics of RepE [54]: (i) *identification*—determine key concepts and find where they are encoded within the model's internal representations (activations or weights); (ii) *manipulation*—measure or quantify the concept's representation and develop techniques to ‘steer’ the model's behaviour; and (iii) *model behaviour*—use manipulated representations to influence the model's output or behaviour on new inputs.

RepE offers a powerful way to understand and control complex AI systems by focusing on their internal representations, offering a more targeted and potentially more effective approach than traditional methods.

### 8.3. Sparse autoencoder models

Sparse autoencoder models (SAM) [56] incorporate a sparsity constraint during training to encourage the network to learn a representation in which only a small number of hidden units are active at any given time. Autoencoders are neural networks trained to reconstruct the inputs. They consist of an encoder (which compresses the input) and a decoder (which reconstructs the input). They learn a compressed, lower-dimensional representation of data, which is often used for dimensionality reduction or feature extraction.

The key characteristics of SAMs are: (i) *autoencoders—*neural networks trained to reconstruct their input, using an encoder and decoder; (ii) *sparsity—a* constraint that forces the network to focus on a smaller set of important features, leading to a more efficient and interpretable representation; and (iii) *efficiency—*by forcing neurons to represent specific features, sparse autoencoders can make the learned representations more interpretable, allowing for a better understanding of the model's inner workings.

Sparse autoencoders are increasingly used to understand the inner workings of LLMs, helping identify and interpret the features learned by these models.

#### 8.3.1. Properties

Finally, table 8 summarizes the properties of the optimization techniques for manipulating the underlying neural network and the associated weights.

## 9. Conclusions—large language model challenges

In summary, LLMs can be categorized in several ways, including their **architecture** (e.g. autoregressive versus encoder-decoder), **access** type (proprietary versus open-source) and **specialization** (general-purpose versus task-specific). We can also categorize LLMs based on their **training** data/regime or primary objectives (e.g. predictive text generation versus instruction following). Consistent with a road-map rather than a benchmark aim, we expect qualitatively: SLMs strong in-domain but narrow; general LLMs broad but hallucination-prone without grounding; reasoning models better on multistep tasks at higher inference cost; agentic systems suited to long-horizon tool-use but prone to cascading errors; and domain-specific models strong in-domain but narrow.

Despite their power, LLMs (transformers) face several key challenges. These include: (i) *computational complexity and resource requirements*—training and deploying large models is extremely costly and slow, and energy consumption is a concern; (ii) *understanding long sequences and dependencies*—even with enhancements, models can struggle with very long-range dependencies or context lengths beyond their window (e.g. maintaining coherence in a long document), which reflects the more fundamental absence of continual learning; (iii) *interpretability and bias*—it remains difficult to interpret why a model produces a given output, and models can inadvertently amplify biases present in training data; and (iv) *optimization challenges*, including fine-tuning for specific tasks without overfitting or losing generality, and ensuring efficient inference (latency, the delay before a response is completed) for real-time applications*.* Additional key areas of concern are aligning models with human values (preventing toxic or harmful outputs) and ensuring that users can trust the model's answers (e.g. distinguishing reliable responses from hallucinations).

The current debate in AI research includes ‘thinking LLMs’ [48]—approaches aimed at improving general AI instruction—by explicitly incorporating internal ‘thought processes’ before generating responses. For example, *thinking LLMs* generate a hidden layer of ‘thoughts’ (an internal chain-of-thought) and refine it through a specialized training method called thought preference optimization (TPO) before producing a final answer. This means that the LLM not only generates an answer in one go; it performs internal reasoning or simulates a thought process, which can lead to more coherent and accurate answers. TPO is a training framework introduced by researchers [48 ] to teach LLMs to ‘think before speaking,’ optimizing the model's internal reasoning via feedback on the final answer quality [48]. Early results from such ‘thinking LLM’ methods show promise across logic, mathematics and even creative tasks, indicating a significant step towards more general and reliable AI systems.

In conclusion, the development of LLMs is a fast-moving field at the intersection of computer science, linguistics and many application domains. Continued innovation in model architectures, training techniques and alignment strategies is critical for addressing current limitations. By systematically tracking these emerging LLM paradigms and understanding their strengths and weaknesses, we can better match LLM technologies to applications that benefit from them, from finance to education, and deploy them in a way that is safe, trustworthy and effective.

## Data Availability

This article has no additional data.
